# THE IMPACT OF RETIREMENT ON VOLUNTEERING FREQUENCY: EVIDENCE FROM A NEW PANEL STUDY

**DOI:** 10.1093/geroni/igz038.470

**Published:** 2019-11-08

**Authors:** Olga Grunwald, Marleen Damman, Kène Henkens

**Affiliations:** 1 NIDI, Den Haag, South Holland, Netherlands; 2 Netherlands Interdisciplinary Demographic Institute, South-Holland, Netherlands

## Abstract

OBJECTIVE: Retirement is a major life transition. People gain a considerable amount of free time, but also loose the benefits from work. Volunteering has been identified as a gratifying way to fill that time and to compensate some retirement-related losses, but is stratified by educational level. Research on how retirement changes volunteering behavior, particularly among different educational groups, is scarce. This study aims to fill this gap. Based on role theory, we hypothesize that the transition into retirement expands levels of volunteering, and that this effect will be relatively strong for the higher educated. METHODS: We use two-wave panel data that were collected in 2015 and 2018 among 5,312 Dutch individuals who were aged 60-65 and employed at baseline. Around half has retired at follow-up (N=2,618). RESULTS: Descriptive findings show that the share of frequent volunteers (i.e., at least once a week) was around 18% at baseline. At follow-up, the share of frequent volunteers rose to 36% among those who retired, but did not change among those who remained employed. Conditional change models show that transitioning into retirement significantly increases volunteering frequency, when controlling for demographic factors and individual resources. As hypothesized, the effect of retirement is relatively strong for the higher educated. DISCUSSION: To deal with the life changes upon retirement, volunteering appears to be an often-used strategy, in particular among the higher educated. Whether this is motivated by work role loss (compensation), or reflects having more time to ‘do good’ (opportunity) is an important question for future research.

